# Large-scale screening of natural genetic resource in the hydrocarbon-producing microalga *Botrycoccus braunii* identified novel fast-growing strains

**DOI:** 10.1038/s41598-021-86760-8

**Published:** 2021-04-02

**Authors:** Koji Kawamura, Suzune Nishikawa, Kotaro Hirano, Ardianor Ardianor, Rudy Agung Nugroho, Shigeru Okada

**Affiliations:** 1 Department of Environmental Engineering, Osaka Institute of Technology, 5-16-1 Ohmiya, Asahi-ku, Osaka, 535-8585 Japan; 2 University of Palangka Raya, Palangkaraya, Indonesia; 3 Faculty of Mathematics and Natural Sciences, Mulawarman University, Samarinda, East Kalimantan Indonesia; 4 Research Center of Natural Products From Tropical Rainforest (PUI PT OKTAL), Mulawarman University, Samarinda, East Kalimantan Indonesia; 5 Graduate School of Agricultural and Life Sciences, The University of Tokyo, Tokyo, Japan

**Keywords:** Applied microbiology, Microbial ecology

## Abstract

Algal biofuel research aims to make a renewable, carbon–neutral biofuel by using oil-producing microalgae. The freshwater microalga *Botryococcus braunii* has received much attention due to its ability to accumulate large amounts of petroleum-like hydrocarbons but suffers from slow growth. We performed a large-scale screening of fast-growing strains with 180 strains isolated from 22 ponds located in a wide geographic range from the tropics to cool-temperate. A fast-growing strain, Showa, which recorded the highest productivities of algal hydrocarbons to date, was used as a benchmark. The initial screening was performed by monitoring optical densities in glass tubes and identified 9 wild strains with faster or equivalent growth rates to Showa. The biomass-based assessments showed that biomass and hydrocarbon productivities of these strains were 12–37% and 11–88% higher than that of Showa, respectively. One strain, OIT-678 established a new record of the fastest growth rate in the race B strains with a doubling time of 1.2 days. The OIT-678 had 36% higher biomass productivity, 34% higher hydrocarbon productivity, and 20% higher biomass density than Showa at the same cultivation conditions, suggesting the potential of the new strain to break the record for the highest productivities of hydrocarbons.

## Introduction

Algal biofuel research aims to make a renewable, carbon–neutral biofuel by using oil-producing microalgae. It initially prospered at 1980th, triggered by oil crisis, and has been reactivated at 2000th by global warming issues^1–3^. At the onset of algal biofuel research in the 1980s, there were large projects to screen natural bioresources, such as the Aquatic Species Program in the USA^4^, and lists of useful oil-producing microalgae were prepared. Subsequently, improved cultivation and harvesting techniques^5,6^, and strain selection and genetic engineering of these algae^7–10^ have been pursued. A high-throughput screening method for lipid-rich microalgae from environmental samples by fluorescence-activated cell sorting is now available^11,12^. A lower-cost direct method of isolation for lipid-rich microalgae using a fluorescence microscope and manipulator has also been reported^13^. This technical progress provides new opportunities to identify cryptic bioresources in nature.

The freshwater, colonial green microalga *Botryococcus braunii* accumulates large amounts of petroleum-like hydrocarbons in the colony^14,15^ and has received much attention in algal biofuel research since most oleaginous microalgae accumulate neutral lipids, which are energetically inferior to hydrocarbons^16,17^. Furthermore, *B. braunii* accumulates the hydrocarbon oils in an extracellular matrix of the colony, which enables “milking” of the algal oils without killing the algae^18,19^. Despite these remarkable characteristics, *B. braunii* has not occupied a leading position in algal biofuel research because of its slow growth rate: typical doubling times are between 3 and 7 days^14,15^. The typical doubling time of *Chlorophyta* is 24 h and that of *Cyanobacteria* is 17 h^20^. The top 20% of *Chlorophyta*, *Cyanobacteria* and other taxa with respect to growth rate have doubling times in the range of 7 to 8 h^20^. Among oleaginous microalgae, *Chlorella vulgaris*, *Neochloris oleoabundans* and *Scenedesmus obliquus* have short doubling times of 8–9 h^21^.

To overcome the slow growth of *B. braunii*, we searched for novel fast-growing strains from natural genetic resources. The Showa strain, known as a fast-growing strain of *B. braunii*, holds the fastest growth record with a doubling time of 1.4 days^22^ and the highest record of hydrocarbon productivity (340 mg L^−1^ d^−1^) to date^23,24^. This strain is a wild strain isolated from the pond of a greenhouse in California in the 1980s and was not selected by screening or artificially modified by mutagenesis^25^. There should be wild strains that grow faster and are more productive than the Showa strain, but, to the best of our knowledge, there has been no large-scale study seeking these strains, a potential of natural genetic resource. A few wild strains and collections of *B. braunii* have been investigated^26–30^, but researchers failed to find significantly faster and more productive strains than Showa, probably due to the limited number of strains tested (< 10) and the limited geographic range of locations searched.

This study performed a large-scale screening of fast-growing strains from a total of 180 strains isolated from 22 ponds located in a wide geographic range, from the tropics to a cool-temperate climate. Although *B. braunii* is widely distributed in freshwater and brackish lakes, reservoirs, or ponds from temperate to tropical environments^15^, their natural densities are commonly quite low (10–10^2^ colonies per L)^31^, which makes it difficult to find the alga in natural environments. We have developed a simple method for isolating *B. braunii* from the natural environments^32^ and have newly isolated 70 wild strains from natural ponds in tropical Indonesia and temperate Japan^31^. This study has isolated an additional 110 strains for the large-scale screening.

The objective of this study is to evaluate the potential of the natural genetic resource of *B. braunii* on an unprecedented scale to answer the following questions with the following approaches: (i) In what kind of natural environment can we expect to find fast-growing strains? We analyzed whether the variations in growth rate between strains are attributable to the differences in the ponds and climate regions where the strains originate. (ii) Are there any wild strains that grow faster than the Showa? We measured biomass and hydrocarbon productivities of nine fast-growing wild strains selected by the screening and compared them to those of Showa and the highest recorded values reported by previous studies for *B. braunii*.

## Results and discussion

### Screening of fast-growing wild strains

**Figure 1:**
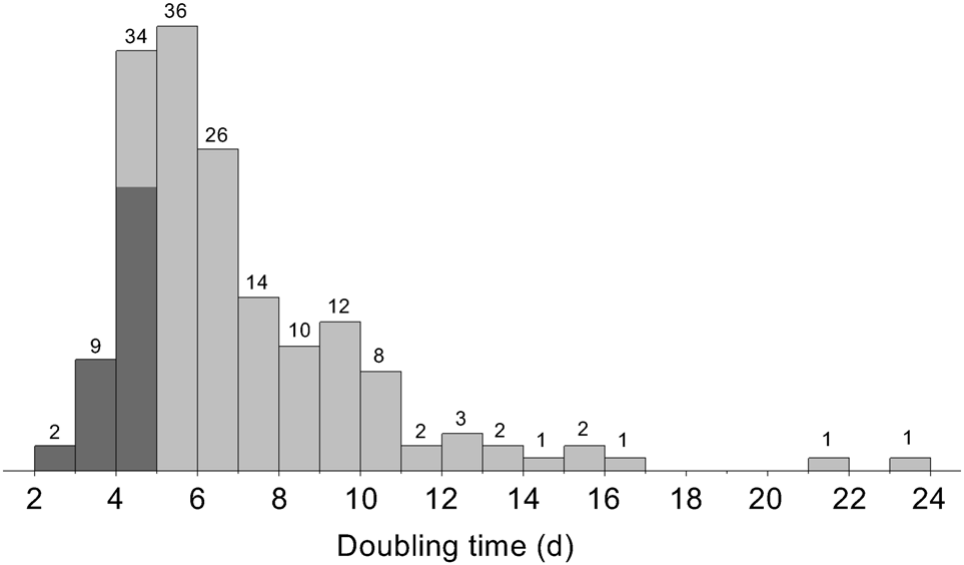
Histogram of doubling time for 163 wild *Botryococcus braunii* strains. Numbers above bars indicate the number of strains. The shaded portion shows wild strains with doubling times shorter than Showa (4.7 days) under the same cultivation conditions.

We have investigated a total of 112 natural ponds, and a total of 180 wild strains were successfully isolated from 22 ponds located in various climate regions (Supplementary Table 1S). The screening of 180 wild strains was performed based on the increasing rate of optical density (OD_660_) in a 10 mL glass tube. The daily increases in OD_660_ in glass tubes were generally well fitted by an exponential function, and the coefficient of determination (*R*^2^) of the curve fitting was > 0.9 for 70% of the data (*n* = 590). After removing the 30% of the data with low *R*^2^ values, we calculated the doubling time (*D*_*t*_) from the exponential growth curve in a total of 163 wild strains. Figure 1 shows the histogram of *D*_*t*_ for 163 wild strains: there was a large variation in *D*_*t*_ with the median at 6.0 days from a minimum of 2.7 days to a maximum of 23.4 days. The Showa strain had a *D*_*t*_ of 4.7 days, and 34 wild strains (20%) had a shorter *D*_*t*_ than Showa. We thus successfully identified several wild strains that are potentially faster-growing than Showa. Of these 34 wild strains, 22 strains (65%) are originated from the tropics (Indonesia). When the chemical races of these fast-growing wild strains were estimated from a molecular phylogenetic analysis of 18S rRNA, 83% were estimated as B race, which produces triterpene hydrocarbons.

**Figure 2:**
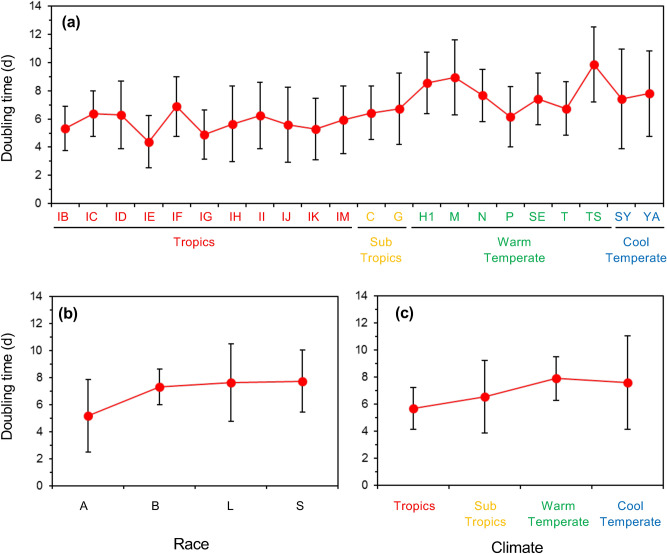
Least square means of the doubling time (*D*_*t*_) of wild strains of *Botryococcus braunii* in relation to chemical races and location of origin. Least square means were computed from a mixed model with race and climate as fixed effects and strain and pond as random effects. Bars indicate 95% confidence intervals. There were no significant differences (*P* > 0.05) between (**a**) Pond, (**b**) Race, or (**c**) Climate. See Supplementary Tables 1S for details of the abbreviation and locations of the ponds.

To answer the question (i) In what kind of natural environment we can expect to find fast-growing strains, we analyzed the relative importance of strain, chemical race, the pond of origin, and climate where the pond is located as determinants of *D*_*t*_ of a strain using a mixed model analysis. The model analysis showed that 57% of the total variance of *D*_*t*_ data was attributable to the variance between strains (σ^2^_*S*_; Table 1). The σ^2^_*S*_ was significantly larger than zero (*P* < 0.05), indicating a significant genetic variation in *D*_*t*_ between strains. In contrast, the variance component of the pond effect (σ^2^_*P*_) was not significantly different from zero (*P* > 0.05). This reflects substantial variations in *D*_*t*_ between strains isolated from the same pond. There were no specific ponds where fast-growing strains originated. The least-square means of *D*_*t*_ calculated from the model for each pond had relatively larger error bars compared to the differences of the least-square means between ponds (Fig. 2). Furthermore, the effects of race and climate on *D*_*t*_ were not significant (*P* > 0.05; Table 2).

**Table 1:** Summary of the fit of a mixed model for doubling-time data of *Botryococcus braunii* wild strains.

Random Effect^a^	Variance ratio	Variance component	SE	95% confidence interval^b^	% of total variance
Strain [Race]	2.62	4.89	0.95	3.03–6.75	57.0
Pond [Climate]	0.97	1.82	1.59	− 1.29 to 4.93	21.2
Residual		1.87	0.16	1.59–2.23	21.8
Total		8.58	1.54	6.21–12.6	100

Model *R*^2^ = 0.79; *n* = 380; *AIC* = 1570; *BIC* = 1609. REML Variance Component Estimates.

^a^Strain was nested in Race, and Pond was nested in Climate.

^b^The lower–upper 95% confidence limits for the variance component. Intervals including zero indicate non-significant variance components (*P* > 0.05).

**Table 2:** Summary of the fit of a mixed model for doubling-time data of *Botryococcus braunii* wild strains.

Fixed effect	No. parameter^b^	DF (DF_Den_)^c^	F Ratio	*P* value
Race^a^	3	3 (39.9)	0.96	0.42
Climate	3	3 (11.6)	1.50	0.27

Model *R*^2^ = 0.79; *n* = 380; *AIC* = 1570; *BIC* = 1609. Fixed Effects Tests.

^a^The Race (A, B, L, S) was estimated based on the molecular phylogeny of 18S rRNA sequences (See Hirano et al.^31^).

^b^Number of parameters associated with the effect.

^c^Degrees of freedom associated with the effect (Denominator degrees of freedom in parenthesis).

Our analyses did not provide any clear answers to the question (i). Fast-growing strains were found in both tropical and temperate climates, and in every pond, there were substantial variations in intrinsic growth rates between strains (Fig. 2). This implies that natural selection does not operate on the growth characteristics measured under laboratory conditions. Because the culture conditions of the screening are far from the conditions in natural environments, the growth rate measured in the laboratory might be a ‘hidden characteristic’ in nature and is unlikely to be exposed to the process of natural selection. Frequent gene flow may also damp the power of natural selection. Hirano et al. reported no genetic differentiation between tropical and temperate strains^31^, probably due to dispersal by birds and wind across a large geographic scale.

### Biomass and hydrocarbon productivities of fast-growing wild strains

**Figure 3:**
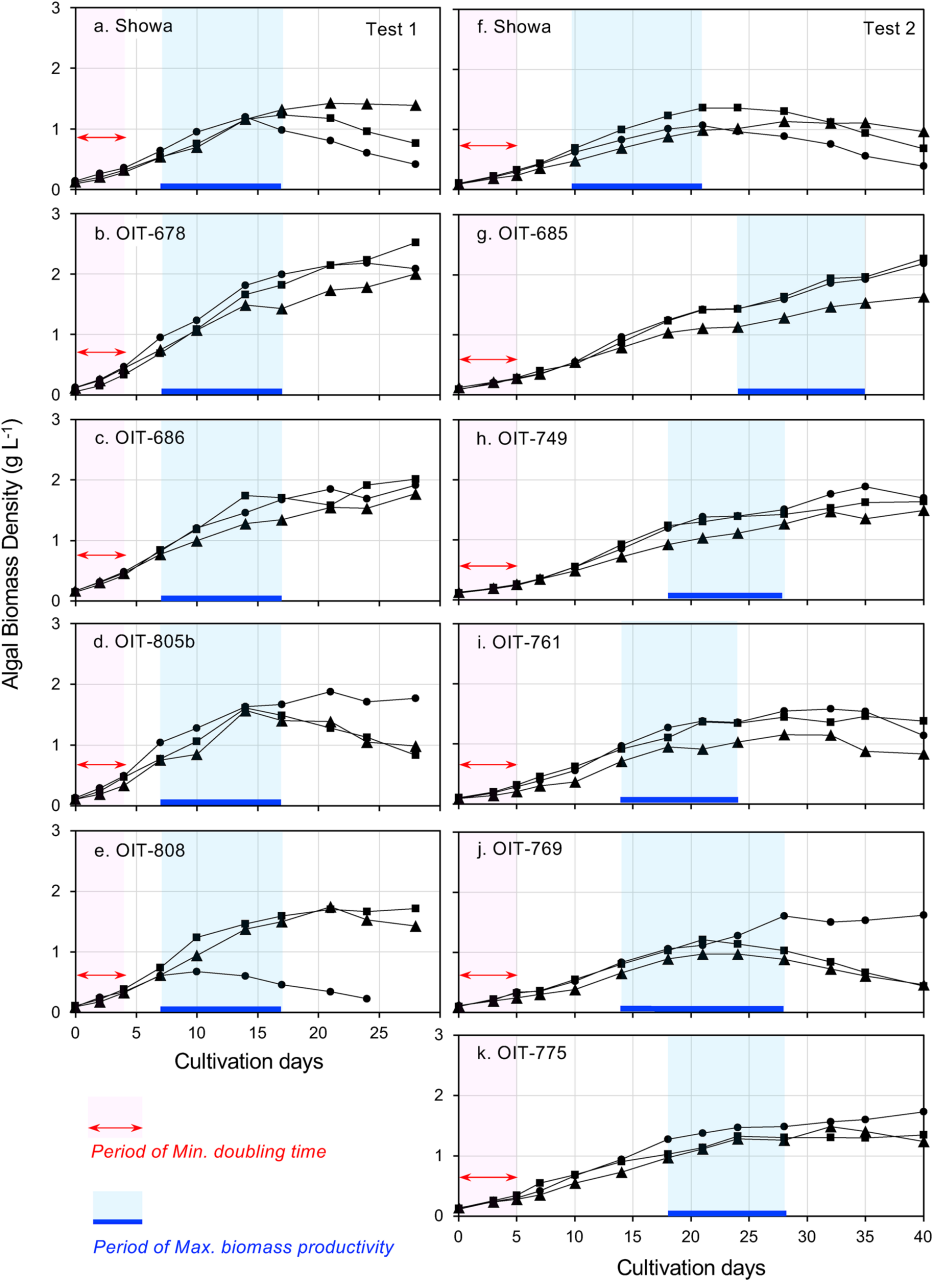
Biomass growth curves of fast-growing wild strains of *Botrycoccus braunii* in a fed-batch culture system. Every 2–3 days, 100 mL (20%) of culture was sampled to estimate algal biomass density, and the same amount of new medium was added. (**a**)–(**e**): Test 1 for 4 wild strains with a standard strain, Showa. (**f**)–(**k**): Test 2 for five wild strains with Showa. There were 3 repetitions (■, ▲, ●) of culture bottles for each strain. The arrow indicates the period of minimum doubling time, the horizontal bar indicates the period of maximum biomass productivity.

We selected 9 fast-growing strains based on the screening results and determined their biomass and hydrocarbon productivities in two separate tests. Test 1 cultured 4 wild strains plus the Showa strain for 30 days, and Test 2 cultured the other 5 strains plus the Showa strain for 40 days. Figure 3 shows the changes in algal biomass density in the fed-batch cultures. Biomass densities increased over the first two weeks, reached a plateau, and then gradually decreased in some culture bottles. The specific biomass growth rate (*μ*_*max*_) was the highest, and *D*_*t*_ was the shortest during the first 4 days of cultivation, and then the *μ*_*max*_ decreased with increasing biomass density. In contrast, biomass productivity (*P*_*x*_) increased with an increase in algal density despite the decrease in the growth rate and reached the maximum at 2–7 days before the peak of algal density.

**Figure 4:**
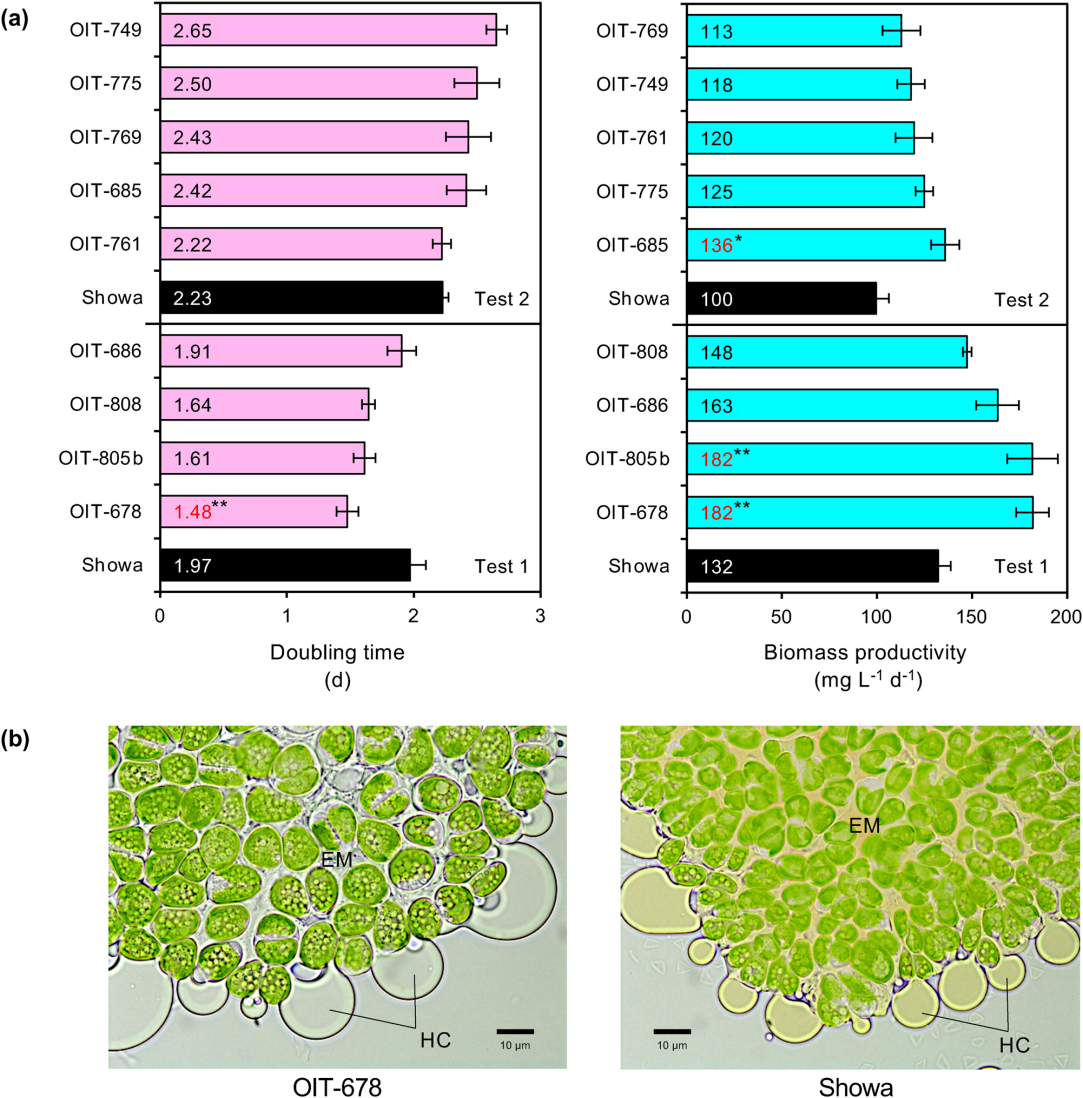
Doubling times and biomass productivity of 9 wild strains and Showa of *Botryococcus braunii*. (**a**) The bar indicates average with standard error (n = 6). Numerical values are shown in the bar. The values significantly different from that of Showa are indicated by asterisks: *, *P* < 0.05; **, *P* < 0.01 (Dunnett’s post hoc test). (**b**) Microscopic view of a wild strain, OIT-678 and the Showa strain. The colony was flattened by cover glass, and hydrocarbons (HCs) exuded from extracellular matrices (EMs).

Figure 4 shows the minimum *D*_*t*_ and the maximum *P*_*x*_ of 9 wild strains compared to the Showa strain. In each culture bottle, the two minimum *D*_*t*_ and the two maximum *P*_*x*_ values were used to calculate the averages for each strain. In Test 1, the *D*_*t*_ of Showa was 1.97 days, and all four wild strains had shorter *D*_*t*_ than Showa. The *D*_*t*_ of OIT-678 (1.48 days) was significantly shorter than that of Showa (*P* < 0.01). In Test 2, another five wild strains had *D*_*t*_ (2.22–2.65 days) similar to that of Showa (2.23 days), and there were no significant differences (*P* > 0.05). These results demonstrate that the first screening by OD measurements in glass tubes successfully identified fast-growing strains with similar or faster biomass growth rates than the Showa strain. The *P*_*x*_ was also significantly higher in fast-growing wild strains than that of Showa (*P* < 0.05, Fig. 4). Three wild strains (OIT-678, OIT-805b, OIT-685) had an approximately 36% higher *P*_*x*_ than Showa (*P* < 0.05). Thus, the fast-growing strains also had high biomass productivities.

Hydrocarbon content, productivity, and constituent were analyzed for 6 productive wild strains (Table 3). The hydrocarbon content of the Showa strain was 26%, and the wild strains had similar or somewhat higher hydrocarbon contents (26–38%). Hydrocarbon productivities of the wild strains ranged from 40 to 58 mg L^−1^ d^−1^, which is 1.1–1.9 times higher than that of Showa. The major constituent hydrocarbons were botryococcenes, C_30_H_50_ for OIT-678, C_33_H_56_ for OIT-805b, C_34_H_58_ for OIT-685, and OIT-775, respectively. Therefore, all these strains were identified as B race.

**Table 3:** Hydrocarbon content, productivity, and major constituent of wild fast-growing *Botryococcus braunii* strains.

Test	Strain	Hydrocarbon content *T*_*HC*_ (%)		Biomass productivity *P*_*x*_ (mg L^−1^ d^−1^)		Hydrocarbon productivity *P*_*HC*_ (mg L^−1^ d^−1^)		Major constituent hydrocarbon
		Mean^a^ (SE)	Ratio	Mean^b^ (SE)	Ratio	a × b	Ratio	
1	Showa	26.6 (1.05)	1	132 (6.6)	1	35	1	NA
	OIT-678	26.0 (0.06)	1.0	182 (8.7)	1.4	47	1.3	C_30_H_50_
	OIT-805b	29.4 (1.55)	1.1	182 (13.3)	1.4	53	1.5	C_33_H_56_
	OIT-686	35.3 (1.45)	1.3	163 (11.3)	1.2	58	1.6	NA
	OIT-808	26.2 (5.11)	1.0	148 (2.3)	1.1	39	1.1	NA
2	Showa	26.4 (0.73)	1	100 (6.7)	1	26	1	NA
	OIT-685	29.2 (3.03)	1.1	136 (7.4)	1.4	40	1.5	C_34_H_58_
	OIT-775	38.9 (4.98)	1.5	125 (4.6)	1.3	49	1.9	C_34_H_58_

Results of two independent experiments, each of which uses Showa strain as a standard, are shown. Ratios of mean values of wild strains to that of the standard strain Showa are also indicated. SE = Standard Error (*n* = 6), NA = Not Analyzed.

### A novel fast-growing strain, OIT-678

**Figure 5:**
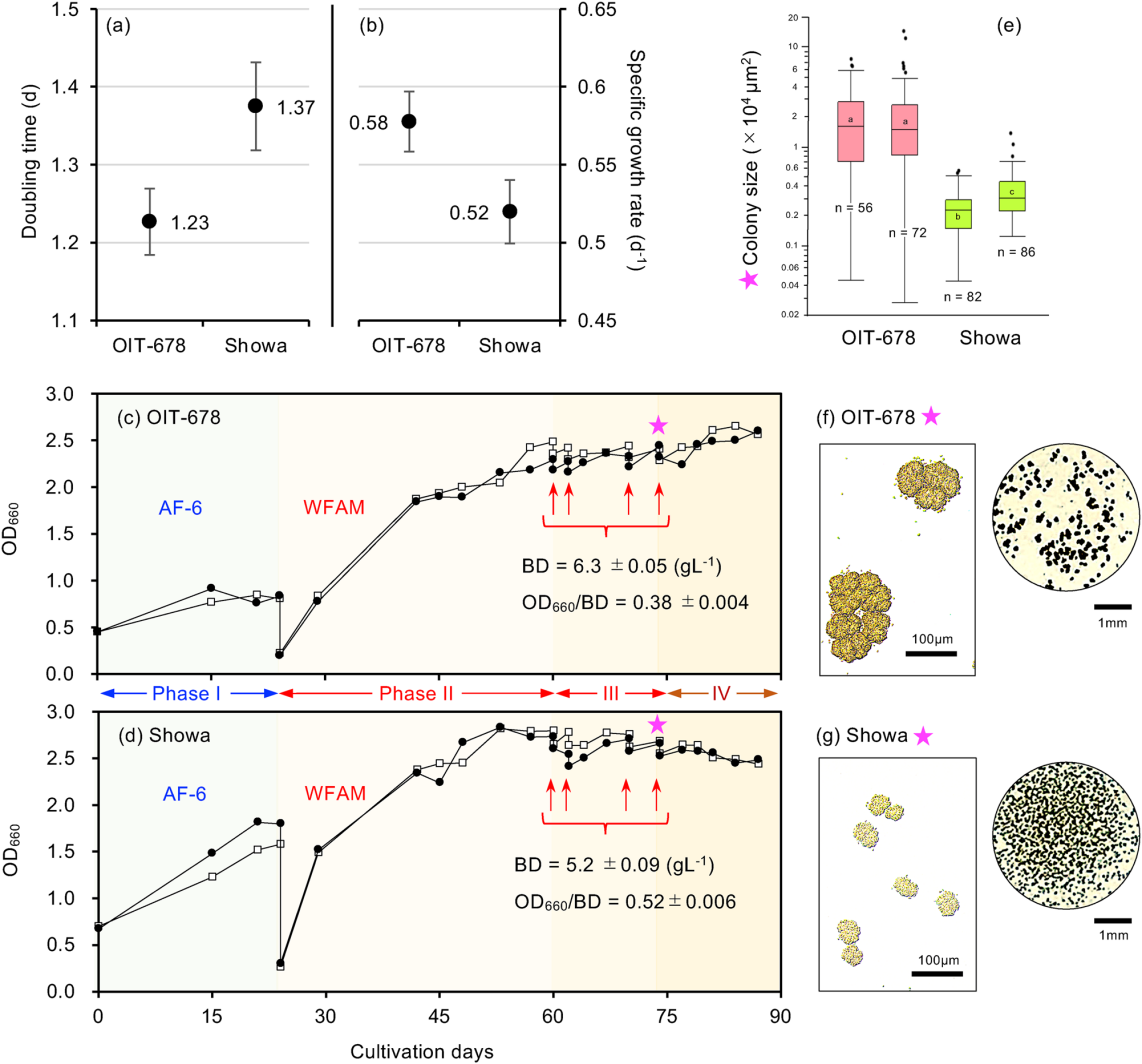
Growth rate, biomass density, and colony size of a novel fast-growing strain, *Botryococcus braunii* OIT-678, compared with the Showa strain. (**a**,**b**) Fast-growing potential was assessed by a 500 mL fed-batch culture (4 replicates for each strain), in which 40% of the culture volume is replaced by new culture medium every 2 days. Until the 10th day of cultivation, biomass growth rates of two consecutive sampling points were calculated, and their averages were compared between strains. (**c**,**d**) Potential for a high-density culture was assessed by using a nutrient-rich medium, WFAM. First, 1 g L^−1^ of algae were inoculated and cultivated in two 500 mL bottles for each strain with AF-6 culture media for 24 days (Phase I). Algal density was monitored by OD. At the end of Phase I, 83 mL of 6 × WFAM were prepared in new 500 mL bottles, and 415 mL of algal culture was transferred to the bottle and cultivated (Phase II). From the 60th to the 75th day, 5% of the algal culture was sampled four times (red arrows), and the same amount of 6 × WFAM was added. This gradually increased the concentration of the media up to 2 × WFAM (Phase III). Cultivation was continued until the 87th day of cultivation (Phase IV). (**e**) Colony size measured on the 75th day (star). The box plot indicates the range of 10–90% of the data with bars and the range of 25–75% of the data with a box. Wilcoxon signed-rank test results are shown by letters in the boxes, and significant differences between the samples are indicated by different letters (*P* < 0.001). (**f**,**g**) Appearance of typical colonies: microscopic view (left) and macroscopic view (right).

The OIT-678 is the only wild strain with a significantly faster growth rate and higher biomass productivity than the Showa strain (Fig. 4). Therefore, we assessed the potential of this strain with additional experiments. To assess the maximal growth rate of the strain, we performed a fed-batch cultivation with a high flow rate (40% replacement every two days). Even in this high-flow rate condition, both OIT-678 and Showa increased biomass density for 10 days. The OIT-678 showed a significantly lower doubling time (t-test: ∣t∣ = 2.1, *df* = 38, *P* < 0.05) and a higher specific growth rate (∣t∣ = 2.06, *df* = 38, *P* < 0.05) than Showa. The *D*_*t*_ of OIT-678 and Showa were 1.23 days and 1.37 days, respectively (Fig. 5a). Although the difference in *D*_*t*_ between the two strains appears small, the outcome in biomass production is quite large. When starting cultivation with the same initial amount of biomass, the biomass of OIT-678 will be twice as much as that of Showa after 12 days. The biomass productivity of OIT-678 was 1.4 times larger than that of Showa, as mentioned earlier (Fig. 4).

We also assessed the ability for long-term, high-density cultivation of the strain by using a nutrient-rich media, WFAM (Supplementary Table 2S). WFAM contains higher amounts of nitrate (841 mg L^−1^ NO_3_^−^) and phosphate (94 mg L^−1^ PO_4_^3−^) than AF-6 (309 mg L^−1^ NO_3_^−^ and 9.7 mg L^−1^ PO_4_^3−^). We started the cultivation by using AF-6 as the culture media and used two 500 mL bottles per strain in batch culture mode (Phase I, Fig. 5c,d). The initial biomass density was adjusted to 1 g L^−1^. After the algal density plateaued on the 24th day of cultivation, the culture media was replaced with WFAM. After replacement, algal density increased again for both strains (Phase II, Fig. 5c,d) and surpassed the plateau of Phase I, suggesting that the increases in algal biomass density were limited by the amounts of nutrients available in Phase I.

A second plateau in the algal density appeared between the 50th and 60th days of cultivation. We then sampled 5% of the algal culture four times (red arrows, Phase III in Fig. 5c,d) and added the same amount of 6 × WFAM. This gradually increased the concentration of the media up to 2 × WFAM until the 75th day of cultivation (Phase III). However, the algal density did not increase for 2 weeks following 75 days of cultivation (Phase IV in Fig. 5c,d), suggesting that the increases in algal biomass density in this phase were light-limited.

The maximal algal biomass density was 6.3 g L^−1^ for OIT-678 and 5.2 g L^−1^ for Showa. OIT-678 had a sixfold larger colony size than Showa (Fig. 5e–g). The increase in colony size induces an aggregated distribution of biomass in the culture and decreases the optical density at a given biomass density^23^. In agreement with this expectation, the ratio of OD to biomass density was smaller in OIT-678 (= 0.38) than in Showa (= 0.52; Fig. 5c,d), indicating a lower light attenuation in the culture of OIT-678. Thus, the OIT-678 can make a higher-density culture than Showa*,* which is at least partly due to the ability to reduce light attenuation in the culture by forming larger colonies.

### Top records of fast growth rate and high productivity

We performed literature reviews on the growth rate and biomass productivity of *B. braunii* to find the fastest growth rates and the highest productivities (Table 4). The OIT-678 strain established a new record of *D*_*t*_ = 1.23 days in *B. braunii* race B, where the previous record was 1.4 days of Showa^22^. The *D*_*t*_ of OIT-678 falls short of the 1.11 days reported in the strain CCAP807/1, race A^27^, and the 0.52 days of an unknown strain^33^. The strain CCAP807/1 showed the *D*_*t*_ of 1.11 days under a continuous light condition (*Php* = 24:0, Table 4), which should be better than our culture condition (*Php* = 14:10). In addition, although the race A and race B strains are classified as the same species, their hydrocarbon structures and biosynthetic processes are largely different^14^, and they showed a high divergence value of 18S rRNA sequences at almost the species level^34^. Therefore, our finding of a novel fast-growing strain in race B is a significant advance.

**Table 4:** Record-holders for the fastest growth rate and highest productivity of biomass and hydrocarbons in *Botycococcus braunii*.

Strain (Race)	Culture conditions					Growth rate		Biomass		Hydrocarbon		*Ref*
	*System*	℃	*PAR*	*Php*	*CO*_*2*_	μ_*max*_	*D*_*t*_	*X*_*max*_	*P*_*x*_	*T*_*HC*_	*P*_*HC*_	
OIT-678 (B)	500-mL flask, semi-continuous	28	100	14:10	2	0.58	1.23	6.2	182	26	47	TS
Showa (B)	30-mL tube, semi-continuous	30	850	14:10	1	0.5	1.40	NA	NA	NA	NA	^22^
Algobank761 (B)	30-mL bubble column, batch	25	100	24:0	4	0.48	1.44	6.4	281	2.0	6	^27^
Showa (B)	0.32-m^2^ trickle-film, continuous	25	282	16:8	5	NA	NA	20	1500	23	340	^23^
AC761 (B)	400-mL bubble column, batch	23	150	18:6	2	0.11	6.3	NA	150	45	68	^26^
CCAP807/1 (A)	30-mL bubble column, batch	25	100	24:0	4	0.624	1.11	8	408	4	15	^27^
UTEX2441 (A)	2-L tubular column, continuous	25	71	NA	NA	0.378	1.83	5.0	223	37	82	^61^
UTEX572 (A)	3.2-L flat PBR, batch	24	300	12:12	NA	NA	NA	7.8	1300	9	110	^35^
CCAP807/1(A)	0.8-L Air-lift cylinder, batch	25	120^a^	14:10	1	NA	NA	2.2	599	44	265	^62^
LB572 (A)	400-mL bubble column, batch	22	50	24:0	2	NA	NA	4.6	296	65^b^	190^b^	^63^
NA	28.57-L flat panel, batch	27	800	NA	1	1.344	0.52	1.8	358	22^b^	80^b^	^33^
TRG (NA)	1.5-L stirred tank, continuous	25	49.5	16:8	NA	0.366	1.89	5.2	368	32	119	^64^

℃, temperature; *PAR*, photosynthetic active radiation (μmols of photons m^−2^ s^−1^); *Php*, photoperiod (light:dark hours); *CO*_*2*_, % v/v; μ_*max*_, specific cell growth rate (d^−1^); *D*_*t*_, doubling time (d); *X*_*max*_, maximum biomass concentration (g L^−1^); *P*_*x*_, biomass productivity (mg L^−1^ d^−1^); *T*_*HC*_, total hydrocarbons (% dry weight); *P*_*HC*_, hydrocarbon productivity (mg L^−1^ d^−1^); *NA*, no information available; *TS*, this study.

^a^Estimated from the value of 26 W m^−2^ of a cold-white fluorescent tube.

^b^Values of total lipids instead of hydrocarbons.

Khichi et al. reported *D*_*t*_ = 0.52 days in a flat panel photobioreactor with an unknown strain of *B. braunii*^33^. This *D*_*t*_ record is extraordinarily fast compared to previous studies. However, they estimated the *D*_*t*_ based on indirect measurements of algal biomass using the OD. The OD of an algal culture can change greatly with the growth of contaminant bacterium as well as changes in the colony size of *B. braunii*^23^. Further studies, including race identification and direct measurements of biomass growth and productivity, are needed to confirm the record as the fastest *B. braunii* strain.

In terms of biomass and hydrocarbon productivity, our wild strains (*P*_*x*_ = 182 mg L^−1^ d^−1^ and *P*_*HC*_ = 58 mg L^−1^ d^−1^; Fig. 4 and Table 3) are not record-breaking. This is because productivity largely changed due to cultivation methods, and our method may not be optimal. Khatri et al. reported an exceptionally large productivity values (*P*_*x*_ = 1500 mg L^−1^ d^−1^, *P*_*HC*_ = 340 mg L^−1^ d^−1^) by an ultra-high density cultivation (*X*_*max*_ = 20 g L^−1^) of the Showa strain (Table 4)^23^, which are some of the highest productivities for algal oil ever reported. In strains belonging to race A, Song et al. also reported a record value of *P*_*x*_ = 1300 mg L^−1^ d^−1^ with a high-density culture (7.8 g L^−1^)^35^. Thus, the high-density culturing appears to be a promising method to improve the volumetric productivities of biomass and hydrocarbons of *B. braunii*. We determined the biomass and hydrocarbon productivities of our wild strains in relatively low densities (0.5–2.0 g L^−1^; Fig. 3). Therefore, there is a great potential for improvement of the productivity of our strains by adopting the high-density cultivation methods.

For high-density cultivations, strains with large-sized colonies might be suitable due to the increased permeability of light. Since light tends to be the most limiting resource for algal growth in high-density cultures, strains with efficient light capture characteristics should be useful. The increase in average colony size in culture is expected to increase the average amount of light received by the surface of a colony because of the reduction of self-shading among colonies^23^. Although the increase in colony size should decrease the amounts of light transmitted into the inner parts of a colony, cells placed in the inner parts of a colony are old and may have a limited physiological capacity to utilize strong light^36^. The OIT-678 strain formed a higher-density culture with a larger-sized colony than Showa (Fig. 5) and is therefore expected to have the potential to achieve higher productivities than Showa in dense-culturing methods.

### Comparisons to other oleaginous microalgae

The growth rates of the Showa strain and OIT-678 are still much slower than those of other fast-growing microalgae. The oleaginous microalgae *Chlorella vulgaris*, *Neochloris oleoabundans*, and *Scenedesmus obliquus* have short doubling times of 8–9 h^21^. Because of its colony-forming habit, *B. braunii* invests resources to construct and maintain the extracellular matrix of the colony, and the self-shading among cells within the colony appears to be unavoidable. The synthesis of energetically expensive hydrocarbons may also restrict the potential for fast growth^14^.

Despite of the slow growth, the biomass and oil productivities of *B. braunii* are comparable to other fast-growing microalgae. The oil productivities of 30 microalgal species, which were assessed in 250 mL flasks under continuous illumination and bubbled with CO_2_-enriched air, showed that the averages (maximums) of the biomass productivity, the lipid content, and the lipid productivity are 190 mg L^−1^ d^−1^ (370 mg L^−1^ d^−1^), 23% (40%), and 40 mg L^−1^ d^−1^ (61 mg L^−1^ d^−1^), respectively^37^. Our novel wild strains of *B. braunii* had comparable productivities (Table 3). Furthermore, a high-density and continuous cultivation of the Showa strain^23^ achieved a hydrocarbon productivity of 340 mg L^−1^ d^−1^, and OIT-678 has the potential to exceed that productivity. This record is one of the highest productivities of algal oil to date. According to a comprehensive review^38^ published in 2011, the highest lipid productivities of microalgae under phototrophic conditions were 142 mg L^−1^ d^−1^ by *Nannochloropsis oculate*^39^ and 133 mg L^−1^ d^−1^ by *Neochloris oleoabundans*^40^. Subsequently, Ho et al. reported a high lipid productivity of 140 mg L^−1^ d^−1^ for *Scenedesmus obliquus*^41^. Further high lipid productivities were then reported for *Chlorella vulgaris* (1425 mg L^−1^ d^−1^)^42^ and *C. protothecoides* (590 mg L^−1^ d^−1^)^43^. Thus, the *Chlorella* species may hold the current record for highest records of lipid productivity of microalgae under phototrophic cultivation.

However, as these records of the *Chlorella* species are instantaneous values measured in batch cultivations, the average productivities under continuous cultivations should be lower than this value. Most oleaginous microalgae, including *Chlorella* and *Scenedesmus* species, require nutrient depletion to initiate lipid accumulation^41–43^. Because the nutrient depletion restricts further biomass growth in the culture, an initial period of cultivation for biomass accumulation is required prior to lipid production. Consequently, even if high lipid productivity is noted during the lipid accumulation phase, the average productivity over the total period, including the period for biomass growth, should decrease^44^. In contrast, *B. braunii* accumulates hydrocarbons mainly during the exponential and early linear growth stages^45–47,62^ (i.e., this alga can produce hydrocarbons and grow biomass simultaneously). In addition, the hydrocarbons produced by *B. braunii* have superior fuel properties to the lipids (triacylglycerol) produced by the oleaginous microalgae such as the *Chlorella* and *Scenedesmus* species in terms of high thermal values and compatibility with the existing petroleum infrastructure. Therefore, *B. braunii* can be regarded as one of the most promising species for the production of algal biofuel, and our novel fast-growing strains are expected to increase the feasibility of biofuel production.

### The promises and challenges of creating a biorefinery from *Botryococcus braunii*

Improvements to cultivation methods such as mixotrophic^48^ and attached cultivation^49^, as well as the high-density cultivation^23^, have great potential to increase the biomass and hydrocarbon productivity of *B. braunii.* Furthermore, biological research is being developed on the genetic transformation^50^, genome sequencing^51^, and bacterial symbionts^52,53^ of *B. braunii*. Although many biotechnological and engineering advances have been made in the course of biomass production and biomass processing techniques to yield biofuels from *B. braunii*, the high production costs are still one of the major issues for the commercialization of algal biofuel production^54^.

In order to reduce the production costs, other potential applications such as wastewater treatment, CO_2_ mitigation, and the manufacture of high-value products should be coupled with biofuel production^2^. Wastewater treatment by *B. braunii* removes nutrients^55^ and heavy metals^56^ and may also be effective for pharmaceutical products remediation as recently reported in other microalgae^57^. Substantial amounts of high-value chemicals aside from hydrocarbons are also identified in *B. braunii*^24,58^. These potential applications can be combined with hydrocarbon production in a biorefinery from *B. braunii*.

When the biorefinery is scaled up to industrial levels in the future, the environmental sustainability concerns of the system cannot be ignored. In particular, due to the irreversibility of energy systems, exergy-based measures should be incorporated into traditional life cycle assessments to analyze the sustainability of the biorefinery from thermodynamic, economic, and environmental perspectives^59^.

### Conclusions

This study performed a large-scale screening of the natural genetic resource of *Botryococcus braunii* on an unprecedented scale with 180 strains isolated from tropical to temperate climates and identified 9 fast-growing strains that have growth rates faster or similar to Showa*,* a standard fast-growing strain. Their biomass productivities were 12–37% higher than that of Showa. One strain, OIT-678, established a new record doubling time (1.2 days) as the fastest race B strain. Further studies are important to test whether the newly-isolated fast-growing strains outperform the highest productivities for hydrocarbons recorded by the formerly fastest strain Showa.

## Methods

### Isolation of wild strains

Since 2015 we have investigated a total of 112 natural ponds, including 50 ponds in tropical Indonesia and 9, 34, and 19 ponds in subtropical, warm-temperate, and cool-temperate Japan, respectively^31,32^. Surface water was collected using a phytoplankton net with 100 μm mesh to capture *B. braunii*, and a single colony was isolated by micropipette and incubated in a glass tube with a culture medium (*See* Kawamura et al.^32^ for a detailed protocol). A total of 180 wild strains were successfully isolated from 22 ponds located in various climate regions (Supplementary Table 1S). The 18S ribosomal sequences were determined for 103 strains, and chemical races (A, B, L, S races)^34^ were estimated based on the molecular phylogenetic tree^31^.

### Screening of fast-growing wild strains

Screening of 180 wild strains was performed based on the growth rate observed in a 10 mL glass tube. The Showa strain was used as a benchmark. The algae were pre-cultured for 2 weeks before screening using AF-6 as the culture media (Supplementary Table 1S) with 100 μmol m^−2^ s^−1^ photosynthetic active radiation (PAR) of 12 h of illumination per day in a 2% CO_2_ incubator at a temperature of 26 ℃. This environmental conditions for cultivation were selected based on the optimal culture conditions for the Showa strain^22^, except for the light intensity (100 μmol m^−2^ s^−1^), which was lower than the optimal (300–1250 μmol m^−2^ s^−1^). This is because in our incubator equipment (*See* Kawamura et al.^32^ for details), strong light illuminations increased temperature (> 30 ℃) and restricted algal growth. The pre-cultured algal cells were transferred into three glass tubes with new culture media by adjusting algal density to be 0.1 of OD_660_. The optical densities (ODs) of the three glass tubes were then measured at 2–3 day intervals for 10–14 days. An exponential curve was fitted to the daily increase in OD, and the specific growth rate and the doubling time were calculated by using Eqs. (1) and (2), respectively.

1 $$OD=k \times {e}^{{(\mu }_{max})\times t}$$

2 $$D_{t}=\frac{\mathrm{ln}2}{{\mu }_{max}}$$

where *μ*_*max*_ = Specific growth rate (d^−1^) and *D*_*t*_ = Doubling time (d).

### Biomass productivity of fast-growing wild strains

Biomass and hydrocarbon productivities were evaluated for a total of 9 wild strains selected by the screening. The selected strains were cultivated in 500 mL glass bottles with AF-6 media by illuminating 100 μmol m^−2^ s^−1^ PAR for 14 h per day with 2% CO_2_ bubbling in an incubator at a constant temperature of 26 ℃. Three culture bottles were used for each strain as a repetition, and the Showa strain was also cultured as a benchmark. Twenty percent of the culture media (100 mL) was sampled every 2 or 3 days, and the culture bottles were replenished with new culture media. At the start of the fed-batch cultivation, 0.1 g L^−1^ algal cells were inoculated. Cultivations were performed for 3–4 weeks. Algal cells were harvested with a 5 μm nylon mesh, dried at 70 ℃ for one week, and algal densities (mg L^−1^) were determined. The biomass productivity (*P*_*x*_) and the specific growth rate (*u*_*max*_) of two consecutive sampling time intervals were calculated by using Eqs. (3) and (4), respectively:

3 $${P}_{x}=\frac{{X}_{t+k}-{0.8\times X}_{t}}{k}$$

4 $${\mu }_{max}=\frac{\mathrm{ln}\left({X}_{t+k}\right)-\mathrm{ln}\left({0.8\times X}_{t}\right)}{k}$$

where *P*_*x*_ = Biomass productivity (mg L^−1^ d^−1^) and *X*_*t*_ = Algal density at the tth days of cultivation (mg L^−1^).

### Hydrocarbon analysis

About 200 mg of the dried algal cells were transferred to a glass tube containing 20 mL of acetone to extract the lipid components, disrupted by ultrasonication for 15 min, and centrifuged at 1000 × *g* for 10 min. The supernatant was transferred to a new glass tube. This process of acetone extraction was repeated 2–3 times until the supernatants became colorless. A chloroform/methanol mixture (25 mL, 2:1, v/v) was then added to the residual biomass, shaken overnight, and filtrated with ADVANTEC 5A filter paper with a vacuum. The filter paper was washed two times in the chloroform/methanol mixture. The chloroform/methanol filtrates and the acetone extracts were combined and concentrated in a rotatory evaporator. The residual lipid component was dissolved in 20 mL *n*-hexane and subjected to silica gel column chromatography on Wakogel C-300 (Wako Pure Chemical Industries, Ltd., Japan) with *n*-hexane as the mobile phase. All eluates before a yellow band of carotenes were collected, evaporated to remove the solvent, dried under vacuum, and then weighed to determine the amounts of hydrocarbons present^22^.

Hydrocarbon compositions were analyzed by gas chromatography/electron impact mass spectroscopy on a Shimadzu QP-2010 Ultra GC/EIMS system (Shimadzu) with a 30 m × 0.25 mm × 0.25 µm Rtx-5 column (GL Sciences). The column temperature was held at 50 °C for 1 min after sample injection, raised to 220 °C at a rate of 10 °C min^−1^ and then to 260 °C at a rate of 2 °C min^−1^ and finally held at 260 °C for 32 min. Splitless injections with a sampling time of 30 s were carried out. Injection temperature was set at 260 °C and injection volume was 2 µl. The ion source was kept at 200 ºC and the temperature of interface was 250 ºC. Identification of hydrocarbons was based on mass spectra and comparisons of retention times with triterpene hydrocarbons previously identified from the B race of *B. braunii*.

### Additional experiments on a novel fast-growing strain, OIT-678

To assess the potential for fast growth of a wild strain (OIT-678), we performed an additional fed-batch cultivation with a high flow rate. The OIT-678 and Showa strains were each cultivated in four 500 mL bottles under the above-mentioned culture conditions for 10 days. During the cultivation, 40% of the culture volume (200 mL) was sampled every 2 days and replenished with new culture media. Specific growth rates were calculated based on the changes in algal density between two consecutive sampling times. A long-term batch culture with nutrient-rich media (WFAM; Supplementary Table 2S) was also performed. Algal density was monitored by OD_660_, and in a stationary phase, 5% of the culture volume (25 mL) was sampled four times to determine the maximal attainable densities. Colony sizes were also measured; microscopic pictures were taken immediately after squashing colonies under a cover glass, and the area (μm^2^) of a colony was measured using Image J software.

### Statistical analyses

We analyzed the relative importance of strain, race, the pond of origin, and climate where the pond is located as determinants of doubling time (*D*_*t*_) of a strain using the model of Eq. (5):

5 $${Y}_{ijklm}=\alpha +{R}_{i}+{C}_{j}+{S}_{\left(i\right)k}+{P}_{\left(j\right)l}+{\varepsilon }_{ijklm}$$

where *Y*_*ijklm*_ is the overall mean *D*_*t*_ estimated in glass tube *m* of stain *k* of race *i,* isolated from pond *l* in climate *j*; α is the overall mean; *R*_*i*_ is the fixed effect of race *i*; *C*_*j*_ is the fixed effect of climate *j*; *S*_*(i)k*_ is the random effect of strain *k* nested in race *i*; *P*_*(j)l*_ is the random effect of pond *l* nested in climate *j*; *ε*_*ijklm*_ is the random residual error. The observed variance (σ^2^_*Y*_) of *D*_*t*_ can be decomposed into the variance of the race effect (σ^2^_*R*_), the variance of the climate effect (σ^2^_*C*_), the variance between the strains within a race (σ^2^_*S*_), the variance between the ponds within climate (σ^2^_*P*_), and the residual error variance (σ^2^_*E*_). The σ^2^_E_ includes the variance between replicated glass tubes within a strain and the error in measurements:

6 $${{\sigma }^{2}}_{Y}={{\sigma }^{2}}_{R}+{{\sigma }^{2}}_{C}+{{\sigma }^{2}}_{S}+{{\sigma }^{2}}_{P}+{{\sigma }^{2}}_{E}$$

Variance components were estimated based on the restricted maximum likelihood (REML) method. The REML method is considered a suitable procedure to estimate variance components for unbalanced data^60^. The least square means of *D*_*t*_ were computed for each strain, race, pond, and climate.

Average specific growth rate, doubling time, and biomass production rate of fast-growing wild strains measured by 500 mL bottle cultivations were compared to those of the Showa strain as a control by using ANOVA followed by Dunnett’s post hoc test. Because colony size data were not normally distributed, the non-parametric Wilcoxon signed-rank test was used instead of ANOVA. All statistical analysis was conducted using JMP software version 8.0 (SAS Institute, Inc., Cary, NC).

## Supplementary Information

Supplementary Information
