# Supplementary material for: Large-scale screening of natural genetic resource in the hydrocarbon-producing microalga Botrycoccus braunii identified novel fast-growing strains
Source: Sci Rep. 2021 Apr 2;11:7368. doi: 10.1038/s41598-021-86760-8 (PMC8018972; doi:10.1038/s41598-021-86760-8)
Supplement: Supplementary file 1 — Supplementary Information [file 41598_2021_86760_MOESM1_ESM.pdf]

Supplementary Information

Scientific Reports

Large-scale screening of natural genetic resource in the hydrocarbon-producing microalga *Botrycoccus braunii* identified novel fast-growing strains

Koji Kawamura*, Suzune Nishikawa, Kotaro Hirano, Ardianor Ardianor, Rudy Agung Nugroho, Shigeru Okada

***Corresponding author**
Koji Kawamura
Email: koji.kawamura@oit.ac.jp
Address: Department of Environmental Engineering, Osaka Institute of Technology, 5-16-1 Ohmiya, Asahi-ku, Osaka, 535-8585 Japan

Table S1. List of wild strains of *Botryococcus braunii* isolated in Japan and Indonesia.

Table S2. Culture media used for algal cultivation.

Table 1S List of wild strains of *Botryococcus braunii* isolated in Japan and Indonesia.

Strain	Pond	Location	Climate	18S rRNA	Race†
OIT-284	SE	Osaka, Japan	Warm Temperate	LC468958	A
OIT-285	SE	Osaka, Japan	Warm Temperate	Unpublished	A
OIT-292	SE	Osaka, Japan	Warm Temperate	LC468959	S
OIT-294	SE	Osaka, Japan	Warm Temperate	Unpublished	A
OIT-297	SE	Osaka, Japan	Warm Temperate	Unpublished	A
OIT-318	N	Fukuoka, Japan	Warm Temperate	LC468960	B
OIT-322	M	Fukuoka, Japan	Warm Temperate	/	/
OIT-323	N	Fukuoka, Japan	Warm Temperate	Unpublished	B
OIT-324	T	Kyoto, Japan	Warm Temperate	Unpublished	B
OIT-326	T	Kyoto, Japan	Warm Temperate	Unpublished	B
OIT-337	C	Okinawa, Japan	Subtropics	Unpublished	B
OIT-340	G	Okinawa, Japan	Subtropics	LC468961	B
OIT-341	C	Okinawa, Japan	Subtropics	Unpublished	B
OIT-345	T	Kyoto, Japan	Warm Temperate	Unpublished	B
OIT-347	G	Okinawa, Japan	Subtropics	LC468962	B
OIT-351	T	Kyoto, Japan	Warm Temperate	LC468963	B
OIT-359	P	Fukuoka, Japan	Warm Temperate	Unpublished	B
OIT-363	P	Fukuoka, Japan	Warm Temperate	Unpublished	A
OIT-369	N	Fukuoka, Japan	Warm Temperate	Unpublished	B
OIT-372	N	Fukuoka, Japan	Warm Temperate	LC468964	B
OIT-373	N	Fukuoka, Japan	Warm Temperate	Unpublished	B
OIT-381	N	Fukuoka, Japan	Warm Temperate	Unpublished	B
OIT-385	N	Fukuoka, Japan	Warm Temperate	Unpublished	B
OIT-395	N	Fukuoka, Japan	Warm Temperate	/	/
OIT-408	T	Kyoto, Japan	Warm Temperate	LC468966	B
OIT-413	T	Kyoto, Japan	Warm Temperate	LC468967	B
OIT-417	T	Kyoto, Japan	Warm Temperate	/	/
OIT-420	G	Okinawa, Japan	Subtropics	Unpublished	B
OIT-431	C	Okinawa, Japan	Subtropics	LC468968	B
OIT-433	C	Okinawa, Japan	Subtropics	/	/
OIT-435	C	Okinawa, Japan	Subtropics	LC468969	B
OIT-439	C	Okinawa, Japan	Subtropics	Unpublished	S
OIT-440	C	Okinawa, Japan	Subtropics	/	/
OIT-446	C	Okinawa, Japan	Subtropics	LC468971	S
OIT-455	C	Okinawa, Japan	Subtropics	/	/
OIT-459	C	Okinawa, Japan	Subtropics	LC468972	B
OIT-460	P	Fukuoka, Japan	Warm Temperate	Unpublished	B
OIT-485	C	Okinawa, Japan	Subtropics	/	/
OIT-486	M	Fukuoka, Japan	Warm Temperate	Unpublished	B
OIT-487	IC	Palangka Raya, Indonesia	Tropics	Unpublished	B
OIT-488	IC	Palangka Raya, Indonesia	Tropics	Unpublished	B
OIT-489	IC	Palangka Raya, Indonesia	Tropics	Unpublished	B
OIT-491	IC	Palangka Raya, Indonesia	Tropics	/	/
OIT-492	IC	Palangka Raya, Indonesia	Tropics	Unpublished	B
OIT-493	IC	Palangka Raya, Indonesia	Tropics	Unpublished	B
OIT-494	IC	Palangka Raya, Indonesia	Tropics	Unpublished	B
OIT-495	IC	Palangka Raya, Indonesia	Tropics	Unpublished	B
OIT-496	IC	Palangka Raya, Indonesia	Tropics	/	/
OIT-498	IC	Palangka Raya, Indonesia	Tropics	LC468974	B
OIT-499	IC	Palangka Raya, Indonesia	Tropics	LC468975	B
OIT-500	IC	Palangka Raya, Indonesia	Tropics	LC468976	B
OIT-501	IC	Palangka Raya, Indonesia	Tropics	Unpublished	B
OIT-502	IC	Palangka Raya, Indonesia	Tropics	LC468977	B

OIT-503	IC	Palangka Raya, Indonesia	Tropics	Unpublished	B
OIT-508	SE	Osaka, Japan	Warm Temperate	LC468978	B
OIT-517	IC	Palangka Raya, Indonesia	Tropics	Unpublished	B
OIT-518	IB	Palangka Raya, Indonesia	Tropics	/	/
OIT-519	IB	Palangka Raya, Indonesia	Tropics	Unpublished	B
OIT-522	IB	Palangka Raya, Indonesia	Tropics	/	/
OIT-536	ID	Palangka Raya, Indonesia	Tropics	LC468979	B
OIT-537	IB	Palangka Raya, Indonesia	Tropics	Unpublished	B
OIT-538	IB	Palangka Raya, Indonesia	Tropics	/	/
OIT-539	ID	Palangka Raya, Indonesia	Tropics	Unpublished	B
OIT-540	IB	Palangka Raya, Indonesia	Tropics	/	/
OIT-541	IB	Palangka Raya, Indonesia	Tropics	Unpublished	B
OIT-542	IB	Palangka Raya, Indonesia	Tropics	Unpublished	B
OIT-543	IB	Palangka Raya, Indonesia	Tropics	Unpublished	B
OIT-544	IB	Palangka Raya, Indonesia	Tropics	/	/
OIT-547	IB	Palangka Raya, Indonesia	Tropics	Unpublished	B
OIT-548	IB	Palangka Raya, Indonesia	Tropics	/	/
OIT-549	IB	Palangka Raya, Indonesia	Tropics	Unpublished	B
OIT-550	IB	Palangka Raya, Indonesia	Tropics	LC468980	B
OIT-551	IB	Palangka Raya, Indonesia	Tropics	/	/
OIT-554	IB	Palangka Raya, Indonesia	Tropics	Unpublished	B
OIT-562	YA	Fukui, Japan	Cool Temperate	LC468982	B
OIT-566	YA	Fukui, Japan	Cool Temperate	/	/
OIT-569	IB	Palangka Raya, Indonesia	Tropics	/	/
OIT-577	IB	Palangka Raya, Indonesia	Tropics	/	/
OIT-578	IB	Palangka Raya, Indonesia	Tropics	Unpublished	B
OIT-579	IB	Palangka Raya, Indonesia	Tropics	/	/
OIT-581	H1	Osaka, Japan	Warm Temperate	LC468983	S
OIT-587	IB	Palangka Raya, Indonesia	Tropics	Unpublished	B
OIT-588	IB	Palangka Raya, Indonesia	Tropics	LC468985	B
OIT-589	IB	Palangka Raya, Indonesia	Tropics	Unpublished	B
OIT-590	IB	Palangka Raya, Indonesia	Tropics	/	/
OIT-591	IB	Palangka Raya, Indonesia	Tropics	/	/
OIT-599	IB	Palangka Raya, Indonesia	Tropics	/	/
OIT-603	IB	Palangka Raya, Indonesia	Tropics	/	/
OIT-605	H1	Osaka, Japan	Warm Temperate	LC468986	S
OIT-611	YA	Fukui, Japan	Cool Temperate	Unpublished	A
OIT-614	YA	Fukui, Japan	Cool Temperate	/	/
OIT-615	YA	Fukui, Japan	Cool Temperate	/	/
OIT-619	IB	Palangka Raya, Indonesia	Tropics	Unpublished	B
OIT-620	H1	Osaka, Japan	Warm Temperate	LC468987	S
OIT-623	SY	Miyagi, Japan	Cool Temperate	LC468988	S
OIT-636	IC	Palangka Raya, Indonesia	Tropics	/	/
OIT-649	IC	Palangka Raya, Indonesia	Tropics	/	/
OIT-652	IC	Palangka Raya, Indonesia	Tropics	/	/
OIT-654	IC	Palangka Raya, Indonesia	Tropics	/	/
OIT-657	IC	Palangka Raya, Indonesia	Tropics	/	/
OIT-660	IC	Palangka Raya, Indonesia	Tropics	/	/
OIT-661	IC	Palangka Raya, Indonesia	Tropics	/	/
OIT-663	IC	Palangka Raya, Indonesia	Tropics	/	/
OIT-668	IC	Palangka Raya, Indonesia	Tropics	/	/
OIT-678	IF	Pundu, Indonesia	Tropics	LC468990	B
OIT-680	IF	Pundu, Indonesia	Tropics	Unpublished	B
OIT-681	IG	Pundu, Indonesia	Tropics	LC468992	B
OIT-682	IG	Pundu, Indonesia	Tropics	/	/
OIT-683	IG	Pundu, Indonesia	Tropics	Unpublished	B

OIT-684	IG	Pundu, Indonesia	Tropics	LC468993	B
OIT-685	IE	Palangka Raya, Indonesia	Tropics	Unpublished	B
OIT-686	IE	Palangka Raya, Indonesia	Tropics	LC468994	B
OIT-696	IC	Palangka Raya, Indonesia	Tropics	/	/
OIT-697	IC	Palangka Raya, Indonesia	Tropics	/	/
OIT-700	IC	Palangka Raya, Indonesia	Tropics	/	/
OIT-701	IC	Palangka Raya, Indonesia	Tropics	/	/
OIT-702	IC	Palangka Raya, Indonesia	Tropics	/	/
OIT-734	H1	Osaka, Japan	Warm Temperate	LC468995	S
OIT-740	TS	Kochi, Japan	Warm Temperate	LC468998	B
OIT-745	IH	Buntok, Indonesia	Tropics	LC468999	L
OIT-749	IE	Palangka Raya, Indonesia	Tropics	Unpublished	B
OIT-750	IE	Palangka Raya, Indonesia	Tropics	LC469001	B
OIT-751	IF	Pundu, Indonesia	Tropics	/	/
OIT-753	IF	Pundu, Indonesia	Tropics	/	/
OIT-756	IF	Pundu, Indonesia	Tropics	Unpublished	B
OIT-757	IF	Pundu, Indonesia	Tropics	/	/
OIT-758	IF	Pundu, Indonesia	Tropics	LC469004	B
OIT-759	IG	Pundu, Indonesia	Tropics	/	/
OIT-760	IG	Pundu, Indonesia	Tropics	LC469005	B
OIT-761	IG	Pundu, Indonesia	Tropics	Unpublished	B
OIT-764	IG	Pundu, Indonesia	Tropics	/	/
OIT-767	IG	Pundu, Indonesia	Tropics	LC469009	B
OIT-768	IG	Pundu, Indonesia	Tropics	/	/
OIT-769	IG	Pundu, Indonesia	Tropics	Unpublished	B
OIT-770	IG	Pundu, Indonesia	Tropics	LC469010	B
OIT-771	IG	Pundu, Indonesia	Tropics	LC469011	B
OIT-772	IG	Pundu, Indonesia	Tropics	/	/
OIT-773	IE	Palangka Raya, Indonesia	Tropics	LC469012	B
OIT-775	IB	Palangka Raya, Indonesia	Tropics	Unpublished	B
OIT-781	II	Muara Teweh, Indonesia	Tropics	LC469014	L
OIT-782	II	Muara Teweh, Indonesia	Tropics	/	/
OIT-784	II	Muara Teweh, Indonesia	Tropics	/	/
OIT-785	II	Muara Teweh, Indonesia	Tropics	LC469015	L
OIT-787	II	Muara Teweh, Indonesia	Tropics	/	/
OIT-788	II	Muara Teweh, Indonesia	Tropics	LC469016	L
OIT-789	II	Muara Teweh, Indonesia	Tropics	/	/
OIT-790	IJ	Palangka Raya, Indonesia	Tropics	Unpublished	B
OIT-798	IK	Palangka Raya, Indonesia	Tropics	LC469020	L
OIT-801	IK	Palangka Raya, Indonesia	Tropics	/	/
OIT-802	II	Muara Teweh, Indonesia	Tropics	/	/
OIT-805b	IE	Palangka Raya, Indonesia	Tropics	Unpublished	B
OIT-808	IE	Palangka Raya, Indonesia	Tropics	LC469023	B
OIT-812	II	Muara Teweh, Indonesia	Tropics	/	/
OIT-817	SE	Osaka, Japan	Warm Temperate	/	/
OIT-821	IK	Palangka Raya, Indonesia	Tropics	/	/
OIT-822	IK	Palangka Raya, Indonesia	Tropics	LC469025	B
OIT-823	IK	Palangka Raya, Indonesia	Tropics	LC469026	L
OIT-824	IK	Palangka Raya, Indonesia	Tropics	LC469027	L
OIT-844	IM	Samarinda, Indonesia	Tropics	Unpublished	B
OIT-848	IM	Samarinda, Indonesia	Tropics	Unpublished	B
OIT-855	IM	Samarinda, Indonesia	Tropics	/	/
OIT-857	IM	Samarinda, Indonesia	Tropics	/	/
OIT-858	IK	Palangka Raya, Indonesia	Tropics	/	/

†, Race was estimated from molecular phylogenetic tree of 18S rRNA.

Table 2S. Culture media used for algal cultivation.

		AF-6†				WFAM§		
NO_3^-		$NaNO_3$	140	mg/L		KNO_3	600	mg/L
		NH_4NO_3	22	mg/L		NH_4NO_3	610	mg/L
PO_4^{3-}		K_2HPO_4	5	mg/L		K_2HPO_4	115	mg/L
		KH_2PO_4	10	mg/L		KH_2PO_4	44.9	mg/L
Mg		$MgSO_4\ 7H_2O$	30	mg/L		$MgSO_4\ 7H_2O$	121	mg/L
						$MgCl_2\ 6H_2O$	103.774	mg/L
Ca		$CaCl_2\ 2H_20$	10	mg/L		$CaCl_2 2H_2O$	129.36	mg/L
Chelate		Na_2EDTA-$2H_2O$	5	mg/L		Fe-Na-$C_{10}H_{14}N_2O_9$	23.1	mg/L
		Fe-citrate	2	mg/L				
		Citric acid	2	mg/L				
Fe		$FeCl_3\ 6H_2O$	0.98	mg/L				
Mn		$MnCl_2\ 4H_2O$	0.18	mg/L		$MnCl_2\ 4H_2O$	0.54	mg/L
Zn		$ZnSO_4\ 7H_2O$	0.11	mg/L		$ZnSO_4\ 7H_2O$	0.066	mg/L
Co		$CoCl_2\ 6H_2O$	0.02	mg/L		$CoSO_4 7H_2O$	0.035	mg/L
Mo		$Na_2MoO_4\ 2H_20$	0.0125	mg/L		$Na_2MoO_4\ 2H_2O$	0.031	mg/L
Cu		*NA*				$CuSO_4\ 5H_2O$	0.0075	mg/L
B		*NA*				H_3BO_3	1.86	mg/L
Buffer		MES	400	mg/L		*NA*		
Vitamin		Biotin	2	µg/L		*NA*		
		Thiamine HCL	10	µg/L		*NA*		
		Vitamin B_6	1	µg/L		*NA*		
		Vitamin B_{12}	1	µg/L		*NA*		

† Media for NIES-collection (https://mcc.nies.go.jp/medium/en/af6.pdf)

§ Khatri *et al.* (2014) *Biotech. Bioeng.*, **111**: 493-503. DOI 10.1002/bit.25126

NA = Not Added.
